# Association between self-reported sleep disorders and prevalence of chronic kidney disease in a nationally representative sample of U.S. adults

**DOI:** 10.3389/fpsyt.2025.1570723

**Published:** 2025-11-18

**Authors:** Hua Xiao, Kai Liu, Guobao Hong, Xinzhou Zhang

**Affiliations:** 1 Department of Nephrology, The Affiliated Shunde Hospital of Jinan University, Foshan, Guangdong, China; 2 Department of Nephrology, Shenzhen People’s Hospital (The Second Clinical Medical College, Jinan University, The First Affiliated Hospital, Southern University of Science and Technology), Shenzhen, Guangdong, China; 3 Department of Intensive Care Unit, The First Affiliated Hospital of Jinan University, Guangzhou, Guangdong, China; 4 Shenzhen Key Laboratory of Kidney Diseases, (The Second Clinical Medical College, Jinan University, The First Affiliated Hospital, Southern University of Science and Technology), Shenzhen, Guangdong, China

**Keywords:** sleep disorders, chronic kidney disease, NHANES, inflammatory markers, chronic non-communicable diseases

## Abstract

**Background:**

Chronic kidney disease (CKD) is associated with various health complications, including sleep disorders. Understanding the relationship between CKD and sleep disturbances is essential for improving patient management and outcomes in this population.

**Patients and methods:**

This study analyzed data from the National Health and Nutrition Examination Survey (NHANES) across two cycles (2015–2016 and 2017-2018), involving 4758 participants aged 20 years and older. Sleep disorders were evaluated via targeted questions from the Sleep Disorders Questionnaire. Chronic kidney disease (CKD) was classified by low estimated glomerular filtration rate (eGFR) and proteinuria. Logistic regression analyses examined the association between sleep disorders and CKD, adjusting for demographic and lifestyle confounders.

**Results:**

Among 4,758 participants, 863 (14%) were diagnosed with chronic kidney disease (CKD). Elevated C-reactive protein (CRP) levels and differential leukocyte counts were observed in the CKD cohort. CKD was associated with increased reports of snoring, sleep apnea, and sleep disturbances, along with higher fatigue levels. A significant positive association between CKD and sleep difficulties persisted in adjusted models. Infrequent snoring (1–2 times per week) demonstrated a negative association, whereas excessive daytime sleepiness (16–30 times/month) correlated positively with CKD. Significant associations between sleep disorders and CKD were observed in participants aged <60, with a BMI <28 kg/m², normal blood pressure, and CRP ≥1.8 mg/L. Sleep disorders were found to be correlated with obesity, hypertension, diabetes, CKD, and proteinuria. Notably, CKD patients with sleep difficulties had markedly elevated CRP levels compared to those without sleep issues, while other inflammatory markers were similarly elevated (P < 0.001).

**Conclusion:**

Patients with chronic kidney disease experience higher rates of sleep issues, highlighting the importance of addressing these problems in CKD management.

## Introduction

Chronic kidney disease (CKD) is currently defined based on a variety of assessed variables indicating abnormalities in kidney structure or function, which include glomerular filtration rate (GFR), urine albumin levels, and the duration of damage (1). It is a common disease worldwide. Due to the rising prevalence of diabetes, hypertension, and obesity, along with the increasing issues of population aging, CKD has reached epidemic proportions. Regardless of the underlying causes, CKD progresses slowly, leading to irreversible loss of nephron units, end-stage renal disease, and premature death (2). According to statistics, in 2017, the global prevalence of CKD reached 9.1%, corresponding to approximately 700 million cases (3, 4). The World Health Organization (WHO) estimates that globally, the annual number of deaths directly attributed to chronic kidney disease ranges from 5 to 10 million, exerting significant pressure on social healthcare resources and individual health (5).

It is generally believed that the circadian rhythm system has evolved as a stabilizing advantage for organisms. However, the lifestyle of modern society, characterized by a 24-hour day and a 7-day week, has made circadian rhythm disruptions increasingly common. Circadian rhythm disruption is defined as a misalignment between the endogenous circadian rhythm and the external environment, with even a one-hour deviation being associated with an increased incidence of cardiovascular events (6). Epidemiological studies in this century indicate that shift workers often experience chronic circadian rhythm disturbances, further increasing the prevalence of chronic kidney disease and hypertension in this population (7–9). Prolonged work hours can also lead to circadian rhythm disruption and are significantly related to declines in kidney function. These data suggest a clear vicious cycle between circadian rhythm disturbances and disease (6). Sleep disorders may manifest as a form of circadian rhythm disruption, as symptoms such as difficulty falling asleep, early waking, and excessive daytime sleepiness may occur when the internal biological clock is misaligned with the external environment or expected schedules.

In current medical research, the interplay between CKD and sleep disorders has garnered increasing attention (10–13). Multiple studies have shown that CKD patients often experience varying degrees of problems with sleep quality, manifesting as insomnia (14–16), sleep apnea (17, 18), and excessive daytime fatigue (11, 19–21). This association is not coincidental, but rather involves complex physiological and pathological mechanisms, in which inflammatory processes may play a crucial role. Therefore, a thorough investigation of the inflammatory pathways associated with sleep disorders in the context of CKD, along with their clinical manifestations, not only aids in elucidating the disease progression but also holds significant practical implications for developing effective intervention strategies to improve the quality of life and prognosis for CKD patients.

## Subjects and methods

### Data source and study population

**Figure 1:**
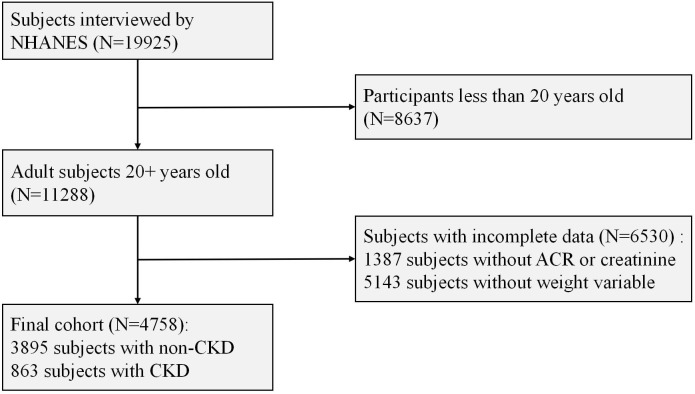
Flow chart of patient selection. Source: NHANES, 2015–2016 and 2017–2018 cycles. NHANES, National Health and Nutrition Examination Survey; CKD, Chronic Kidney Disease; ACR, Albumin-to-Creatinine Ratio.

The data for this study was derived from the National Health and Nutrition Examination Survey (NHANES). NHANES is a cross-sectional survey conducted by the National Center for Health Statistics under the Centers for Disease Control and Prevention (CDC). This survey received approval from the Institutional Review Board of the CDC, and written informed consent was obtained from all participants. A stratified, random, multi-stage probability cluster design was employed to select households that were interviewed, aiming to monitor trends in the health and nutritional status of the non-institutionalized civilian population in the United States. NHANES is a publicly available dataset with ongoing updates. To enhance research accuracy and reduce sampling bias, data from two cycles (2015–2016 and 2017-2018) of NHANES were merged. Participants aged 20 years and older were included in the study (n=8637). Individuals lacking data on serum creatinine and urine albumin-to-creatinine ratio were excluded (n=1387), as were those with missing fasting glucose weight variables (n=5143). After screening, a total of 4758 participants were incorporated into this analysis, of which 14% (n=863) were identified as CKD patients (**Figure 1**).

### Assessment of sleep disorders

While numerous health and lifestyle factors may contribute to circadian rhythm disturbances, for the purposes of this study, the assessment of sleep disorders was streamlined by utilizing specific items from the “Sleep Disorders Questionnaire.” The following questions were evaluated: “Ever told doctor had trouble sleeping”, “Sleep hours”, “How often do you snore?”, “How often do you snort or stop breathing?”, and “How often feel overly sleepy during day?” “Sleep hours” is categorized into three groups: long sleepers (>9 hours), normal sleepers (6–9 hours), and short sleepers (<6 hours).

### Definition of low eGFR, proteinuria, and chronic kidney disease

The primary outcome of our study was to assess the chronic kidney disease status of the participants, which we defined as either a low estimated glomerular filtration rate (eGFR, ml/min/1.73 m²) or the presence of proteinuria (1). Laboratory data were collected for serum creatinine levels, measured using the Jaffe reaction enzymatic method, for both serum and urine samples. eGFR (22) was calculated using the Chronic Kidney Disease Epidemiology Collaboration (CKD-EPI) equation based on demographic data and serum creatinine levels. Participants with an eGFR of less than 60 mL/min/1.73 m² were classified as having low eGFR. Urinary albumin data were also obtained from laboratory measurements, utilizing solid-phase fluorescent immunoassay methods. Proteinuria was defined as a urinary albumin-to-creatinine ratio (UACR) of 30 mg/g or greater. It is important to note that low eGFR and proteinuria can exert independent effects on the body. Therefore, our secondary outcome included the assessment of participants for the presence of either proteinuria or low eGFR.

### Demographic variables

Demographic variables were obtained through standardized questionnaires administered during the interviews. The following variables were recorded: age, gender (male and female), race/ethnicity (Mexican American, Other Hispanic, Non-Hispanic White, Non-Hispanic Black, Non-Hispanic Asian, Other/Multiracial), marital status (married, widowed, divorced, separated, never married and living with a partner), Ratio of family income to poverty (PIR), and education level (Less than 9th grade, 9th-11th grade, high school diploma/GED or equivalent, some college or AA degree, college or higher). The PIR was utilized to assess household income. Lifestyle factors included body mass index (BMI), smoking status, diabetes, and hypertension. BMI was evaluated in the Mobile Examination Center (MEC) and categorized into non-obesity (<30 kg/m²) and obesity (≥30 kg/m²). Smoking status was classified into two categories: never smokers and current smokers. Never smokers were defined as individuals who have smoked no more than 100 cigarettes in their lifetime, while current smokers were defined as those who had smoked at least 100 cigarettes. Diabetes was defined based on one of the following criteria: self-reported history of diabetes, currently taking insulin or diabetes medication, fasting glucose level ≥7.0 mmol/L (126 mg/dL), or hemoglobin A1c (HbA1c) level ≥6.5%. Hypertension was defined based on blood pressure measurements and medication use, with categories classified as normal, prehypertension, and hypertension. Normal blood pressure was defined as systolic blood pressure (SBP) <120 mmHg and diastolic blood pressure (DBP) <80 mmHg without the use of antihypertensive medications. Prehypertension was defined as SBP between 120–139 mmHg or DBP between 80–89 mmHg without the use of antihypertensive medications. Participants with SBP ≥140 mmHg, DBP ≥90 mmHg, or those reporting current use of antihypertensive medications were classified as having hypertension. In cases where systolic and diastolic pressures fell into different categories, participants were assigned to the more severe category.

### Systemic inflammatory markers

Systemic inflammatory markers were assessed using neutrophil, monocyte, eosinophil, and total white blood cell counts (measured in 10³ cells/µL) alongside C-reactive protein (CRP) levels (measured in mg/L). The white blood cell counts were conducted using the Beckman Coulter DxH-800 Analyzer, while CRP levels were measured using the Roche Cobas 6000 (c501 module).

### Statistical analysis

Statistical analyses were conducted using R software (version 4.2.1) following the NHANES data analysis guidelines. The study design accounted for survey design factors, non-response, and post-stratification, assigning sample weights to each participant to consider survey sampling weights. For continuous variables and those following a normal distribution, means and standard deviations were calculated. Descriptive statistics and Chi-square tests were performed to evaluate differences in the distribution of categorical variables. Independent samples t-tests or non-parametric tests were utilized to assess mean differences in continuous variables across groups. Logistic regression analyses were employed to explore the association between CKD and sleep disorders, with odds ratios (ORs) and 95% confidence intervals (CIs) reported to adjust for potential confounders. A two-tailed P-value of less than 0.05 was considered statistically significant. Three distinct logistic regression models examine the association between different types of sleep disorders and CKD status, with each sleep disorder included as an independent variable and CKD status as the dependent variable. In addition to an unadjusted Model 1, Model 2 included gender and race to account for sociodemographic factors, while Model 3 incorporated gender, race, BMI, smoking status, education level, and PIR. Sensitivity analyses were performed on a dataset that had not been adjusted for weight variables. Subgroup analyses were carried out to examine the associations between sleep disorders and CKD, low eGFR, and proteinuria within various subgroups, stratified by age (<60 years and ≥60 years), BMI (<28 kg/m² and ≥28 kg/m²), blood pressure status (hypertension/normal blood pressure/prehypertension), CRP (<1.8 mg/L and ≥1.8 mg/L), and PIR (<2.88 and ≥2.88). Adjustments were made for gender, race, BMI, smoking status, education level, and PIR. Additionally, we employed logistic regression models, treating sleep disorders as independent variables and chronic non-communicable diseases as dependent variables, incorporating multiple covariates such as gender, race, BMI, smoking status, education level, and PIR to examine the association between sleep disorders and chronic non-communicable diseases.

## Results

### Baseline characteristics of the study population

Among 4,758 participants, 863 (14%) were diagnosed with CKD. The CKD group comprised 57% females and 43% males, with an average age of 65 years compared to 45 years in the non-CKD group. The average BMI was 29 kg/m² in CKD patients and 28 kg/m²in non-CKD individuals. There were also significant differences in marital status, education level, and family poverty level between the two groups. CKD patients exhibited a higher prevalence of diabetes and hypertension. CKD patients had elevated CRP levels. Significant differences in white blood cell counts, monocyte counts, neutrophil counts, and eosinophil counts were observed, with higher counts in CKD patients. In the CKD cohort, the snoring frequency, occurrence of sleep apnea, and reporting of sleep disturbances to a physician were all significantly higher compared to the non-CKD group. Additionally, CKD patients reported a higher frequency of feeling fatigue (**Table 1**).

**Table 1:** Baseline data of study population for participants with and without CKD from the national health and nutrition examination survey, 2015-2016 and 2017-2018 cycles.

Characteristic	Overall, N=4758(100%)	No CKD, N=3895(86%)	CKD, N=863(14%)	P-value
Sex				0.036
Female	2,465 (52%)	2,019 (51%)	446 (57%)	
Male	2,293 (48%)	1,876 (49%)	417 (43%)	
Age (year)	48 (33, 61)	45 (32, 58)	65 (52, 76)	<0.001
Race				0.086
Mexican American	738 (9.0%)	610 (9.1%)	128 (8.7%)	
Other Hispanic	559 (6.7%)	482 (7.0%)	77 (5.3%)	
Non-Hispanic White	1,601 (63%)	1,254 (62%)	347 (65%)	
Non-Hispanic Black	1,030 (11%)	832 (11%)	198 (13%)	
Non-Hispanic Asian	616 (5.7%)	542 (6.0%)	74 (4.2%)	
Other/multiracial	214 (4.5%)	175 (4.6%)	39 (3.7%)	
Marital status				<0.001
Married	2,415 (54%)	2,000 (54%)	415 (49%)	
Widowed	340 (5.3%)	199 (3.5%)	141 (16%)	
Divorced	549 (11%)	432 (11%)	117 (13%)	
Separated	167 (2.3%)	134 (2.2%)	33 (2.4%)	
Never married	843 (18%)	743 (19%)	100 (11%)	
Living with partner	442 (9.8%)	387 (9.9%)	55 (8.9%)	
Education level				<0.001
Less than 9th grade	477 (5.1%)	364 (4.7%)	113 (7.6%)	
9-11th grade	566 (8.3%)	442 (7.9%)	124 (11%)	
High school graduate/GED or equivalent	1,090 (25%)	861 (24%)	229 (31%)	
Some college or AA degree	1,463 (31%)	1,220 (32%)	243 (29%)	
College graduate or above	1,161 (30%)	1,008 (32%)	153 (22%)	
BMI (kg/m(2))	28 (25, 33)	28 (24, 33)	29 (25, 35)	0.006
Smoked at least 100 cigarettes in life	2,072 (44%)	1,642 (44%)	430 (50%)	0.018
Ratio of family income to poverty	2.88 (1.52, 5.00)	2.97 (1.57, 5.00)	2.43 (1.41, 4.18)	0.003
Diabetes				<0.001
Yes	1,078 (16%)	679 (12%)	399 (41%)	
No	447 (9.0%)	381 (8.9%)	66 (9.4%)	
Uncertain	3,233 (75%)	2,835 (79%)	398 (50%)	
Doctor told you have diabetes				<0.001
Yes	761 (11%)	453 (8.1%)	308 (30%)	
No	3,854 (87%)	3,330 (90%)	524 (66%)	
Borderline	140 (2.3%)	109 (2.0%)	31 (4.2%)	
Taking insulin now	227 (2.8%)	103 (1.8%)	124 (8.7%)	<0.001
Take diabetic pills to lower blood sugar	622 (44%)	386 (39%)	236 (57%)	<0.001
Fasting glucose (mmol/L)	5.66 (5.27, 6.22)	5.61 (5.27, 6.11)	6.11 (5.55, 7.38)	<0.001
Glycohemoglobin (%)	5.50 (5.20, 5.80)	5.40 (5.20, 5.70)	5.90 (5.50, 6.60)	<0.001
Hypertension				<0.001
Hypertensive	2,005 (38%)	1,386 (32%)	619 (71%)	
Normotensive	1,428 (37%)	1,331 (40%)	97 (15%)	
Prehypertensive	1,073 (25%)	957 (27%)	116 (14%)	
Systolic (mmHg)	120 (112, 132)	118 (110, 130)	132 (118, 146)	<0.001
Diastolic (mmHg)	70 (64, 78)	70 (64, 78)	70 (62, 78)	0.9
Taking prescription for hypertension	1,605 (89%)	1,078 (87%)	527 (95%)	0.003
Albumin creatinine ratio (mg/g)	7 (4, 12)	6 (4, 9)	46 (16, 106)	<0.001
Creatinine (mg/dL)	0.83 (0.70, 0.97)	0.82 (0.70, 0.95)	0.96 (0.73, 1.20)	<0.001
CRP (mg/L)	1.8 (0.8, 4.3)	1.7 (0.7, 4.1)	2.3 (1.0, 5.4)	<0.001
WBC (10^3^cells/uL)	6.50 (5.50, 7.90)	6.40 (5.50, 7.80)	6.90 (5.70, 8.60)	<0.001
Monocyte (10^3^cells/uL)	0.50 (0.40, 0.60)	0.50 (0.40, 0.60)	0.60 (0.50, 0.70)	<0.001
Neutrophils (10^3^cells/uL)	3.74 (2.90, 4.80)	3.70 (2.90, 4.70)	4.20 (3.20, 5.25)	<0.001
Eosinophils (10^3^cells/uL)	0.20 (0.10, 0.30)	0.20 (0.10, 0.20)	0.20 (0.10, 0.30)	0.002
Sleep hours (hour)				<0.001
<6h	469.00 (7.78%)	377.00 (7.37%)	92.00 (10.34%)	
>=6h and <=9h	3,725.00 (82.48%)	3,110.00 (83.89%)	615.00 (73.68%)	
>9h	534.00 (9.74%)	390.00 (8.74%)	144.00 (15.98%)	
How often do you snore				<0.001
Never	1,134 (25%)	919 (24%)	215 (29%)	
Rarely - 1-2 nights a week	1,121 (29%)	971 (30%)	150 (18%)	
Occasionally - 3-4 nights a week	871 (19%)	731 (20%)	140 (18%)	
Frequently - 5 or more nights a week	1,301 (27%)	1,027 (26%)	274 (34%)	
How often do you snort or stop breathing				<0.001
Never	3,318 (75%)	2,767 (76%)	551 (72%)	
Rarely - 1-2 nights a week	602 (13%)	501 (14%)	101 (10%)	
Occasionally - 3-4 nights a week	295 (6.4%)	233 (6.2%)	62 (7.7%)	
Frequently - 5 or more nights a week	247 (4.8%)	183 (4.0%)	64 (9.8%)	
Ever told doctor had trouble sleeping	1,365 (31%)	1,061 (30%)	304 (39%)	<0.001
How often feel overly sleepy during day				0.014
Never	827 (13%)	704 (13%)	123 (12%)	
Rarely - 1 time a month	1,115 (23%)	936 (24%)	179 (21%)	
Sometimes - 2-4 times a month	1,607 (36%)	1,327 (37%)	280 (33%)	
Often- 5-15 times a month	811 (19%)	637 (19%)	174 (22%)	
Almost always - 16-30 times a month	393 (7.9%)	290 (7.3%)	103 (12%)	
Low eGFR	375 (6.0%)	0 (0%)	375 (43%)	<0.001
Albuminuria	637 (9.8%)	0 (0%)	637 (70%)	<0.001
CKD	863 (14%)	0 (0%)	863 (100%)	<0.001

Continuous variables were reported as means ± SD. Categorical variables were reported as N (%).

BMI, Body Mass Index; CRP, C-Reactive Protein; WBC, White Blood Cell; GED, General Educational Development; AA, Associate of Arts degree; eGFR, estimated Glomerular Filtration Rate; CKD, Chronic Kidney Disease.

### Relationship between sleep disorders and CKD

A positive association exists between CKD and reporting sleep difficulties, which remains significant in adjusted models. Compared to individuals with normal sleep duration, both long sleepers (>9 hours) and short sleepers (<6 hours) are associated with a higher incidence of CKD. There is a negative association with infrequent snoring (1–2 times per week), while no significant association is noted for frequent snoring (≥5 times per week). Additionally, less frequent sleep apnea (1–2 times per week) demonstrated no significant association, but a positive association was observed for more frequent instances (≥5 times per week). Lastly, CKD correlates positively with excessive daytime sleepiness occurring 16–30 times per month (**Table 2**).

**Table 2:** Association between sleep characteristics and chronic kidney disease.

Characteristic	Model 1		Model 2		Model 3	
	OR, (95%CI)	P value	OR, (95%CI)	P value	OR, (95%CI)	P value
Ever told doctor had trouble sleeping						
No						
Yes	1.05 (1.02,1.09)	0.002	1.05 (1.02,1.08)	0.005	1.04 (1.01,1.08)	0.025
Sleep hours						
>=6h and <=9h						
<6h	1.06 (1.00, 1.12)	0.037	1.06 (1.01, 1.12)	0.031	1.05 (0.98, 1.13)	0.2
>9h	1.11 (1.04, 1.18)	0.003	1.11 (1.04, 1.18)	0.003	1.09 (1.02, 1.17)	0.016
How often do you snore						
Never						
Rarely - 1-2 nights a week	0.93 (0.90,0.96)	<0.001	0.93 (0.90,0.96)	<0.001	0.92 (0.89,0.96)	0.001
Occasionally - 3-4 nights a week	0.97 (0.93,1.01)	0.091	0.97 (0.93,1.01)	0.14	0.96 (0.92,1)	0.042
Frequently - 5 or more nights a week	1.01 (0.97,1.05)	0.7	1.01 (0.97,1.06)	0.5	0.99 (0.94,1.04)	0.7
How often do you snort or stop breathing						
Never						
Rarely - 1-2 nights a week	0.97 (0.94,1.01)	0.2	0.98 (0.94,1.02)	0.2	0.97 (0.92,1.01)	0.12
Occasionally - 3-4 nights a week	1.03 (0.97,1.1)	0.3	1.04 (0.97,1.11)	0.2	1.03 (0.96,1.11)	0.4
Frequently - 5 or more nights a week	1.16 (1.04,1.29)	0.008	1.16 (1.04,1.29)	0.01	1.14 (1.02,1.28)	0.026
How often feel overly sleepy during day						
Never						
Rarely - 1 time a month	1.00 (0.95,1.04)	0.8	0.99 (0.95,1.03)	0.6	1.01 (0.96,1.07)	0.7
Sometimes - 2-4 times a month	1.00 (0.95,1.05)	>0.9	0.99 (0.95,1.04)	0.8	1.01 (0.96,1.07)	0.7
Often- 5-15 times a month	1.03 (0.98,1.08)	0.2	1.02 (0.98,1.07)	0.3	1.04 (0.98,1.1)	0.2
Almost always - 16-30 times a month	1.08 (1.01,1.16)	0.022	1.07 (1.00,1.15)	0.042	1.07 (0.99,1.16)	0.089

Model 1: No adjust;

Model 2: Adjusted for sex, race;

Model 3: Adjusted for sex, race, BMI, Smoked at least 100 cigarettes in life, Education level, Ratio of family income to poverty.

OR, odds ratio.

Source: NHANES, 2015–2016 and 2017–2018 cycles.

### Subgroup analysis of sleep disorders related to CKD, low eGFR, and proteinuria

**Figure 2:**
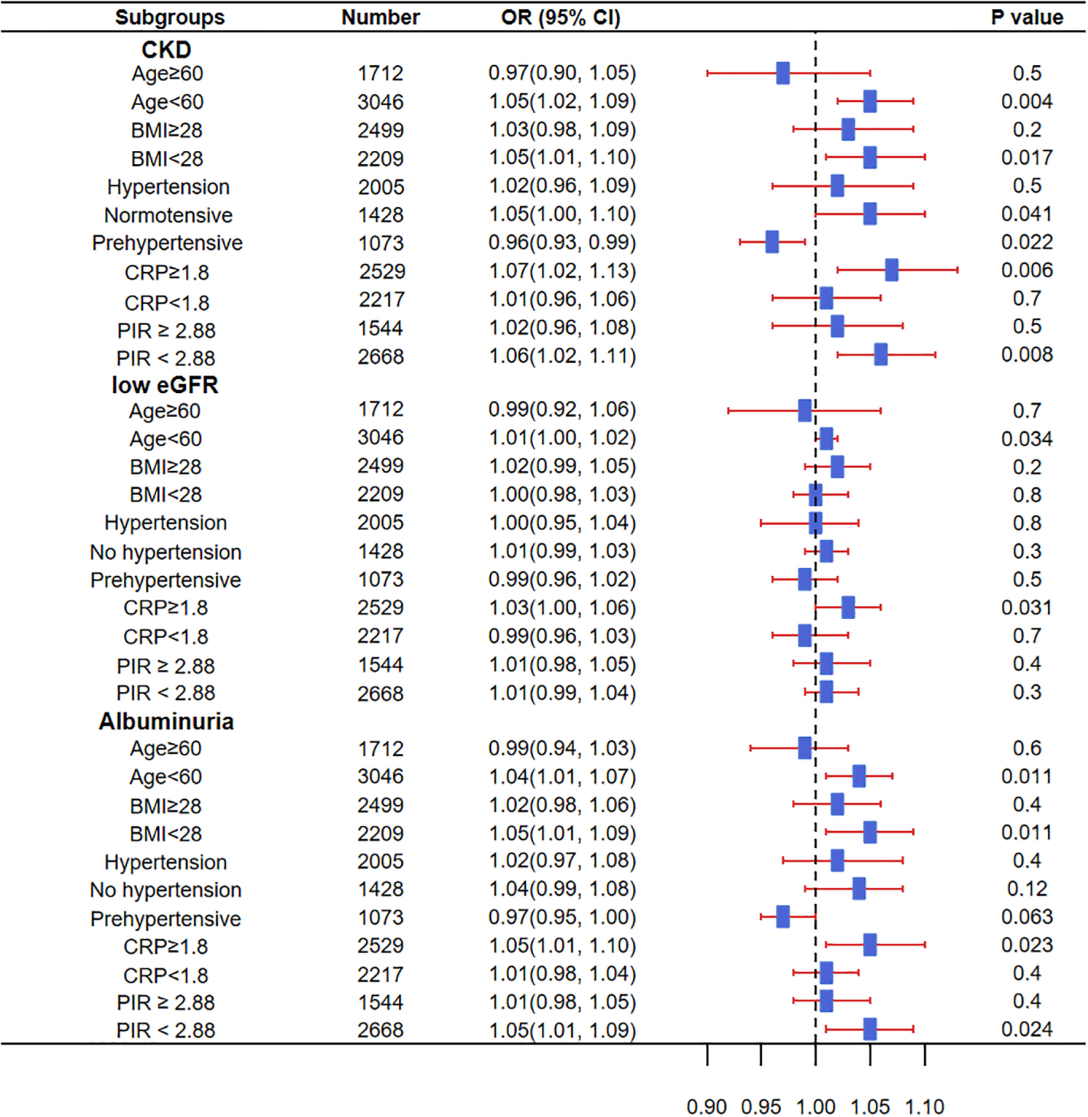
Impact of sleep disorders in different subgroups on the prevalence of CKD, low eGFR, and proteinuria. OR, Odds Ratio; CKD, Chronic Kidney Disease; BMI, Body Mass Index; CRP, C-Reactive Protein; PIR, Ratio of family income to poverty; eGFR, estimated Glomerular Filtration Rate. Source: NHANES, 2015–2016 and 2017–2018 cycles.

Significant associations were found between sleep disorders and CKD in participants aged <60 years, BMI <28 kg/m², normal blood pressure, CRP ≥1.8 mg/L, and PIR <2.88. In pre-hypertensive participants, sleep disorders showed a negative association with CKD. Additionally, sleep disorders were significantly associated with low eGFR in those aged <60 years and CRP ≥1.8 mg/L. Furthermore, significant associations with proteinuria were observed in participants aged <60 years, BMI <28 kg/m², CRP ≥1.8 mg/L, and a household income-to-poverty ratio <2.88 (**Figure 2**).

### Association between sleep disorders and chronic non-communicable diseases

**Figure 3:**
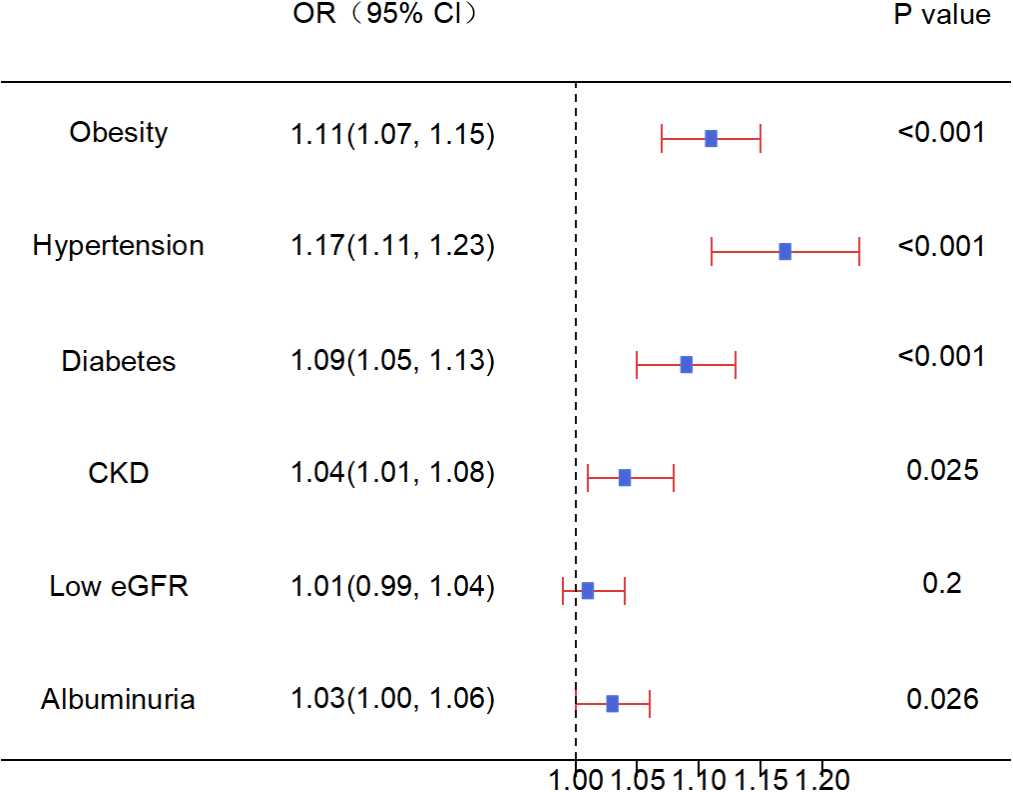
Association between sleep disorders and chronic non-communicable diseases. OR, Odds Ratio; CKD, Chronic Kidney Disease; eGFR, estimated Glomerular Filtration Rate. Source: NHANES, 2015–2016 and 2017–2018 cycles.

Sleep disorders in adults were found to be correlated with obesity, hypertension, diabetes, CKD, and proteinuria, but no association was observed with low eGFR (**Figure 3**).

### Relationship between sleep disorders and blood biomarkers in CKD patients

In CKD patients, those reporting sleep difficulties had a higher average CRP level compared to those without sleep issues. No significant differences were observed in average white blood cell count, monocyte count, neutrophil count, and eosinophil count. In participants with sleep difficulties, CKD patients had a significantly higher CRP level than non-CKD participants. CRP levels, average white blood cell count, monocyte count, and neutrophil count were markedly higher in CKD patients with sleep difficulties than in CKD-free individuals (P < 0.001), while eosinophil count showed no significant difference (**Table 3**).

**Table 3:** Association between sleep disorders in CKD patients and blood biomarkers.

Characteristic	CKD and trouble sleeping, N = 304 (5.4%)	Non-CKD and trouble sleeping, N = 1061 (25%)		CKD and non-trouble sleeping, N = 558 (8.5%)		Non-CKD and non-trouble sleeping, N = 2833 (61%)	
	Mean (SD)	Mean (SD)	*P*-value	Mean (SD)	*P*-value	Mean (SD)	*P*-value
CRP (mg/dL)	6.7 ± 15.2	4.1 ± 6.9	<0.001	4.3 ± 8.2	0.002	3.5 ± 6.4	<0.001
WBC (10^3^ cells/uL)	7.38 ± 2.19	7.01 ± 2.3	0.046	7.33 ± 3.9	0.2	6.71 ± 1.89	<0.001
Monocyte (10^3^ cells/uL)	0.6 ± 0.19	0.56 ± 0.18	0.057	0.59 ± 0.2	0.4	0.54 ± 0.17	<0.001
Neutrophils (10^3^ cells/uL)	4.52 ± 1.72	4.13 ± 1.75	0.001	4.33 ± 1.57	0.085	3.89 ± 1.5	<0.001
Eosinophils (10^3^ cells/uL)	0.23 ± 0.18	0.2 ± 0.15	0.2	0.24 ± 0.18	0.6	0.2 ± 0.15	0.14

CKD, Chronic Kidney Disease. SD, standard deviation; CRP, C-Reactive Protein; WBC, White Blood Cell;

Source: NHANES, 2015–2016 and 2017–2018 cycles.

## Discussion

The ICSD-3 diagnostic criteria for sleep disorders systematically analyze 241 distinct diagnostic criteria and categorize them into nine types, including clinical manifestations, objective markers, distress, and disability (23). Sleep disorders encompass a wide range of conditions, from obstructive sleep apnea to rapid eye movement sleep behavior disorder. In the diagnosis of sleep disorders, doctors can initially determine the presence of a sleep disorder by thoroughly understanding the patient’s medical history, including sleep patterns, daytime dysfunction, and related symptoms. Traditional polysomnography is considered the gold standard for diagnosing sleep disorders. It can record multiple physiological signals, including electroencephalography, electrooculography, and electromyography, thereby providing a comprehensive assessment of the various stages and quality of sleep (24). Self-assessment scales and questionnaire tools play an important role in the preliminary screening and assessment of sleep disorders. These tools typically include standardized scales, such as the Pittsburgh Sleep Quality Index and the Epworth Sleepiness Scale, which can help doctors quickly evaluate patients’ sleep quality and daytime sleepiness levels. The data in this article comes from the NHANES database, with participants being a large civilian population. The assessment of sleep disorders in this study is based on questionnaire surveys. Although lacking the precision of polysomnography or activity monitors, this method provides a cost-effective screening approach, which is particularly valuable in large-scale observational studies.

CKD and sleep disorders influence each other. Patients with CKD often have hyperphosphatemia and hypocalcemia, and these electrolyte changes may lead to increased neural excitability, thereby affecting the normal sleep cycle (21). The accumulation of toxins in the body of patients with uremia can have a negative impact on the central nervous system, leading to issues such as insomnia, daytime sleepiness, and sleep apnea (25). Uremia may also cause inflammatory responses in the central nervous system, which are considered an important physiological mechanism leading to sleep disorders (26). Sleep disorders in CKD patients are also associated with an increased risk of cardiovascular disease. There is a bidirectional relationship between cardiovascular disease and CKD, where cardiovascular issues may further exacerbate the severity of sleep disorders, creating a vicious cycle. Common symptoms of sleep apnea in CKD patients are closely related to the occurrence of cardiovascular events (10). Sleep disorders such as insomnia and circadian rhythm disturbances lead to reduced or disrupted secretion of melatonin (27, 28). Melatonin has antioxidant, anti-inflammatory, and anti-fibrotic effects, and its deficiency can exacerbate oxidative stress and fibrosis in the kidneys (29, 30). Sleep disorders lead to sustained sympathetic nervous system hyperactivity and parasympathetic nervous system inhibition. The sympathetic nervous system is crucial for maintaining renal function homeostasis. Excessive sympathetic nerve activity can cause functional and morphological changes in renal physiology and structure, potentially resulting in kidney damage and the progression of chronic kidney disease. Muscle sympathetic nerve activity and norepinephrine show a significant negative correlation with renal function indicators (31, 32). Parasympathetic inhibition weakens anti-inflammatory functions, such as the cholinergic anti-inflammatory pathway, exacerbating renal inflammation (33, 34). These pathophysiological mechanisms involve bidirectional pathways. The data in this article comes from the NHANES database and is a cross-sectional study, which cannot determine the temporal sequence between CKD and sleep disorders. Although reverse causation cannot be ruled out, statistical methods have been employed to control for key confounding factors. Future assessments are needed to conduct longitudinal studies and interventional trials focusing on sleep treatment in patients with CKD.

The main innovation of our research lies in the comprehensive assessment of the association between various sleep disorders and the chronic kidney disease using the NHANES dataset, which provides a robust and representative sample of the U.S. population. Previous studies have primarily focused on specific sleep disorders, such as sleep apnea or insomnia, within chronic kidney disease populations, often limited to small cohorts or specific subgroups (35, 36). Our research integrates various sleep disorders, including sleep duration, snoring frequency, sleep apnea, and daytime excessive sleepiness, to examine their association with chronic kidney disease. Furthermore, our study is innovative in adjusting for multiple confounding variables, including demographic factors, lifestyle behaviors, and systemic inflammation markers. This comprehensive approach allows us to gain a more detailed understanding of the associations between various sleep disorders and chronic kidney disease. The research indicates that sleep apnea is associated with the progression of chronic kidney disease. Our findings reveal that intermittent sleep apnea and excessive sleepiness significantly associated with chronic kidney disease. In addition, we provided key insights using logistic regression and subgroup analysis, revealing how these associations vary across different demographic and health characteristics. We found interesting phenomena, particularly that younger individuals and those with lower BMI or higher CRP levels are especially susceptible to the adverse effects of sleep disorders on kidney function. This level of detail enhances the clinical relevance of our findings, suggesting targeted interventions for specific high-risk populations. Clinicians should consider incorporating sleep assessments into the routine care plans for patients with chronic kidney disease to identify and manage these disorders early.

This study has several limitations that should be acknowledged. First, the cross-sectional design of the NHANES data prevents establishing a causal relationship between sleep disorders and CKD. Although we can identify associations, we cannot infer causality from this study design. Second, relying on self-reported data for sleep disorders and other lifestyle factors introduces potential recall bias and misclassification. Participants might not accurately recall or report their sleep patterns and other health behaviors, which can impact the reliability of the findings. Furthermore, the wide heterogeneity among sleep disorders, ranging from obstructive sleep apnea to REM sleep behavior disorder, each with distinct pathophysiological mechanisms, could also impact the interpretability and robustness of the observed associations. Moreover, the absence of objective measures, such as actigraphy or polysomnography, further constrains the accuracy of sleep disorder diagnoses. CKD was treated as a binary outcome, without considering disease progression or severity. This approach may overlook potential differences in the association between sleep disorders and CKD across various stages of disease severity. Lastly, while the study controlled for a variety of potential confounders, residual confounding cannot be entirely ruled out. Future research should aim to use longitudinal designs to better assess causality, incorporate objective measures of sleep disorders, and ensure a more representative sample to enhance the generalizability of the findings.

## Conclusion

CKD patients had a higher prevalence of sleep difficulties reported to physicians, higher CRP levels, and increased counts of white blood cells, monocytes, neutrophils, and eosinophils compared to non-CKD participants. CKD is positively associated with both short and long sleep durations, frequent sleep apnea (≥5 times per week), and excessive daytime sleepiness (16–30 times per month), while infrequent snoring (1–2 times per week) shows a negative association, and less frequent sleep disturbances generally lack significant associations. Subgroup analysis revealed significant associations between sleep disorders and CKD in specific populations.

## Data Availability

Publicly available datasets were analyzed in this study. This data can be found here: The data can be directly accessed through the NHANES website: NHANES Data.
